# Hierarchical and homotopic correlations of spontaneous neural activity within the visual cortex of the sighted and blind

**DOI:** 10.3389/fnhum.2015.00025

**Published:** 2015-02-10

**Authors:** Omar H. Butt, Noah C. Benson, Ritobrato Datta, Geoffrey K. Aguirre

**Affiliations:** ^1^Department of Neurology, University of PennsylvaniaPhiladelphia, PA, USA; ^2^Department of Psychology, University of PennsylvaniaPhiladelphia, PA, USA

**Keywords:** spontaneous activity, blindness, resting state fMRI, homotopic correlation, functional connectivity mapping

## Abstract

Spontaneous neural activity within visual cortex is synchronized by both monosynaptic, hierarchical connections between visual areas and indirect, network-level activity. We examined the interplay of hierarchical and network connectivity in human visual cortex by measuring the organization of spontaneous neural signals within the visual cortex in total darkness using functional magnetic resonance imaging (fMRI). Twenty-five blind (14 congenital and 11 postnatal) participants with equally severe vision loss and 22 sighted subjects were studied. An anatomical template based on cortical surface topology was used for all subjects to identify the quarter-field components of visual areas V1-V3, and assign retinotopic organization. Cortical visual areas that represent the same quadrant of the visual field were considered to have a hierarchical relationship, while the spatially separated quarters of the same visual area were considered homotopic. Blindness was found to enhance correlations between hierarchical cortical areas as compared to indirect, homotopic areas at both the level of visual areas (*p* = 0.000031) and fine, retinotopic scale (*p* = 0.0024). A specific effect of congenital, but not postnatal, blindness was to further broaden the cortico-cortico connections between hierarchical visual areas (*p* = 0.0029). This finding is consistent with animal studies that observe a broadening of axonal terminal arborization when the visual cortex is deprived of early input. We therefore find separable roles for vision in developing and maintaining the intrinsic neural activity of visual cortex.

## Introduction

Synchronized neural activity between brain regions creates correlations in functional magnetic resonance imaging (fMRI) signals (Friston, 1994; Biswal et al., 1995; Greicius et al., 2003). The form and distribution of functional correlations, also known as “resting state functional connectivity,” reflects both the hierarchical, monosynaptic connections between brain areas (Hagmann et al., 2008; Skudlarski et al., 2008) and the ongoing, dynamic pattern of neural activity in the system (Damoiseaux and Greicius, 2009; Honey et al., 2009).

Within the human visual system, structured correlations have been observed in spontaneous signals collected at rest that could reflect direct anatomical connections, the indirect effects of network activity, or both. The visual cortex is composed of multiple, retinotopic visual areas (Engel et al., 1994; Sereno et al., 1995; DeYoe et al., 1996; Dumoulin and Wandell, 2008). Monosynaptic connections couple retinotopically-aligned neurons across visual areas (Bauer et al., 1999; Lyon and Kaas, 2002). Functional MRI correlations between sequential cortical areas in the visual pathway have been reported at the level of visual areas (Liu et al., 2007) and fine-scale retinotopic organization (Heinzle et al., 2011; Raemaekers et al., 2013), and could include a contribution from monosynaptic, “hierarchical” connections.

Functional correlations are also found between areas of the visual cortex that are not directly connected. Most prominently, there is synchronization of neural signals across the visual cortex at locations with similar retinotopic representations of visual field eccentricity (Bao and Tjan, 2009; Heinzle et al., 2011; Yeo et al., 2011; Raemaekers et al., 2013; Buckner and Yeo, 2014; although the signal may not reflect eccentricity *per se*, see Butt et al., 2013). This signal extends across visual areas and hemispheres of the brain and does not correspond to any known structural, white matter pathway (Manger et al., 2002; Jeffs et al., 2009; Innocenti and Price, 2005). These “homotopic” correlations (Jo et al., 2012) presumably arise from the pattern of synchronous activity across a network of brain areas that share common afferents and efferents (Adachi et al., 2012).

Here, we examine how blindness interacts with these two components of functional correlation. We examined fMRI resting state correlations between hierarchical and homotopic pairs of visual areas in 22 sighted and 25 blind people while they rested in darkness. The blind population included 14 subjects with congenital blindness and 11 with postnatal blindness (onset after age seven), allowing us to dissociate the early developmental effects of blindness from the ongoing effect of loss of visual input. This correlation structure was examined both at the level of entire visual areas (as has been previously studied by Liu et al., 2007; Yu et al., 2008; Watkins et al., 2012; and Burton et al., 2014) and at the fine-scale of points on the cortical surface with matched eccentricity representations.

## Materials and methods

### Subjects

Forty-seven subjects participated (Table S1; 28 females and 19 males), 25 with vision loss, and 22 normally sighted controls. The blind participants (mean age of 54) included 14 congenital and 11 postnatally blind. The 22 sighted subjects (mean age of 37) had normal or corrected-to-normal visual acuity. As normal aging is associated with changes in resting state correlations (Zuo et al., 2010; Ferreira and Busatto, 2013), we identified sub-groups of 15 subjects each with similar mean ages (47 for the blind group, 44 for the sighted group). Participants were screened to exclude those who had recently taken cyclooxygenase-1 inhibitors (e.g., ibuprofen), as these compounds can alter neuro-vascular coupling (Bruhn et al., 2001). Handedness was assessed using a standard questionnaire (Bryden, 1982). The study was approved by the University of Pennsylvania Institutional Review Board, and all subjects provided informed consent and were compensated for their time. A prior report of these data restricted the analysis to area V1 (Butt et al., 2013).

### Full-field visual threshold

Full-field visual threshold (FST) represents the minimum achromatic light intensity threshold a dark-adapted subject can perceive. FST was determined for 14 of the 16 blind patients with residual light perception; two subjects declined FST testing. Following pharmacologic pupil dilation and 20 min of dark adaptation, subjects were studied using an Espion Full-Field ERG system supplemented with a full field visual threshold determination module running Espion Diagnosys V5.0.34 software. Each eye was tested sequentially and the best performance (lowest threshold) retained. The intensity of a flashed light (4 ms, 6500 K) was varied on each of many trials during which the subject indicated by button press if a given stimulus was seen. The stimulus intensity was increased and then decreased over multiple staircase runs until a 50% response threshold was identified, typically after 225 trials. Thresholds were expressed in decibels (dB) relative to the 0.01 cd· s/m^2^ starting point, with normal performance being between −35 and −50 dB (Messias et al., 2013).

### Magnetic resonance imaging

A 3-Tesla Siemens Trio with an 8-channel Siemens head coil was used to collect echoplanar BOLD fMRI data with whole brain coverage (TR = 3 s; 3 × 3 × 3 mm isotropic voxels; TE = 30 ms; FA = 90°; 42 axial slices, interleaved; 64 × 64 voxel in-plane resolution; no parallel acceleration). Head motion was minimized with foam padding. Functional scans were obtained in total room darkness and stray light was minimized using opaque shades and covering light sources. Subjects were instructed to close their eyes during the scan, hold still, and stay awake. No other instructions were given and subjects were not exposed to any other time varying stimulus. 160 TRs were collected for 9 subjects and 150 TRs for 38 subjects; only the first 150 TRs of data were used in all cases. Continuous pulse-oximetry was recorded for 40 of 47 scanning sessions. One or more anatomical images using a standard T1-weighted, high-resolution anatomical scan of Magnetization Prepared Rapid Gradient Echo (3D MPRAGE) (160 slices, 1 × 1 × 1 mm, TR = 1.62 s, TE = 3.09 ms, TI = 950 ms, FOV = 250 mm, FA = 15°) were acquired for each subject.

### Image pre-processing

Anatomical data from the subjects were processed using the FMRIB Software Library (FSL) toolkit (http://www.fmrib.ox.ac.uk/fsl/) to correct for spatial inhomogeneity and to perform non-linear noise-reduction. Brain surfaces were reconstructed and inflated from the MPRAGE images using the FreeSurfer (v5.1) toolkit (http://surfer.nmr.mgh.harvard.edu/) as described previously (Dale et al., 1999; Fischl and Dale, 2000; Salat et al., 2004).

Following sinc interpolation in time to correct for slice acquisition order and six parameter, least-squares motion correction, the echoplanar data were co-registered to subject specific anatomy in FreeSurfer using FSL-FLIRT with 6 degrees-of-freedom under a FreeSurfer wrapper (bbregister). Serial whole brain (right & left hemisphere) surface maps for each individual TR were transformed from volumetric to surface space using nearest neighbor interpolation (point sampling) of the midpoint position between the white and pial surfaces in Freesurfer using mri_vol2surf, before being registered to a common FreeSurfer template surface pseudo-hemisphere (fsaverage_sym) using the FreeSurfer spherical registration system (Fischl et al., 1999; Greve et al., 2013).

The surface-sampled raw data were smoothed using a 2 mm full-width at half-max, 2-dimensional Gaussian kernel. Modest spatial smoothing increases the sensitivity and specificity of the final correlation structure (Vincent et al., 2006), while decreasing spatial noise (Greicius et al., 2003). A 0.01–0.08 Hz bandpass filter was applied to the time-series (Biswal et al., 1995; Yeo et al., 2011). Nuisance covariates were removed, including spikes (periods of raw signal deviation greater than two standard deviations from the mean), head-motion as modeled by three translation and three rotation covariates, mean deep white-matter and ventricular signal, and physiologic signals (when available). The latter were derived from raw, 50 Hz pulse oximetry measurements taken during BOLD scanning. After aligning raw pulse data with DICOM image time-stamps, raw pulse arrays were split into high (cardiac) and low (respiratory) frequency covariates (Verstynen and Deshpande, 2011). Global signal covariates were not included (Saad et al., 2012), although a nuisance covariate derived from the mean deep white-matter and ventricular signal was included (Jo et al., 2010). The inclusion of this last nuisance covariate was the only difference in data pre-processing from the analysis performed in the prior report of these data (Butt et al., 2013).

### Visual cortex mean surface area, thickness, and deformation values

The cortical surface area (mm^2^) was obtained as the area enclosed by vertices comprising V1, V2, and V3 cortex at the gray-white matter boundary. The mean segmented gray-matter thickness was obtained for each region and scaled by the mean cortical thickness for the entire brain. The mean areal deformation warp for the visual cortical region on the white surface was quantified by obtaining the log of the determinant of the Jacobian matrix (Fischl et al., 2001) for all vertices. The standard deviation of these values may be taken as a “goodness of fit” for registration of the cortical surface anatomy to the anatomical template.

### Phantom scans

The component of hierarchical and homotopic spatial correlation induced by MR image properties and data processing was estimated from eight functional scans of a Siemens cylindrical phantom (1900 mL, 115 mm diameter × 200 mm, distilled water with 3.75 g NiSO_4_/ 1000 g H_2_O). The analyses from eight randomly selected subjects were repeated exactly but supplied with the phantom data containing 150 volumetric image time-series. Measurements of hierarchical and homotopic correlation in the phantom at both the level of visual areas and fine-scale were obtained.

### Calculating whole-region functional correlation matrices

**Figure 1:**
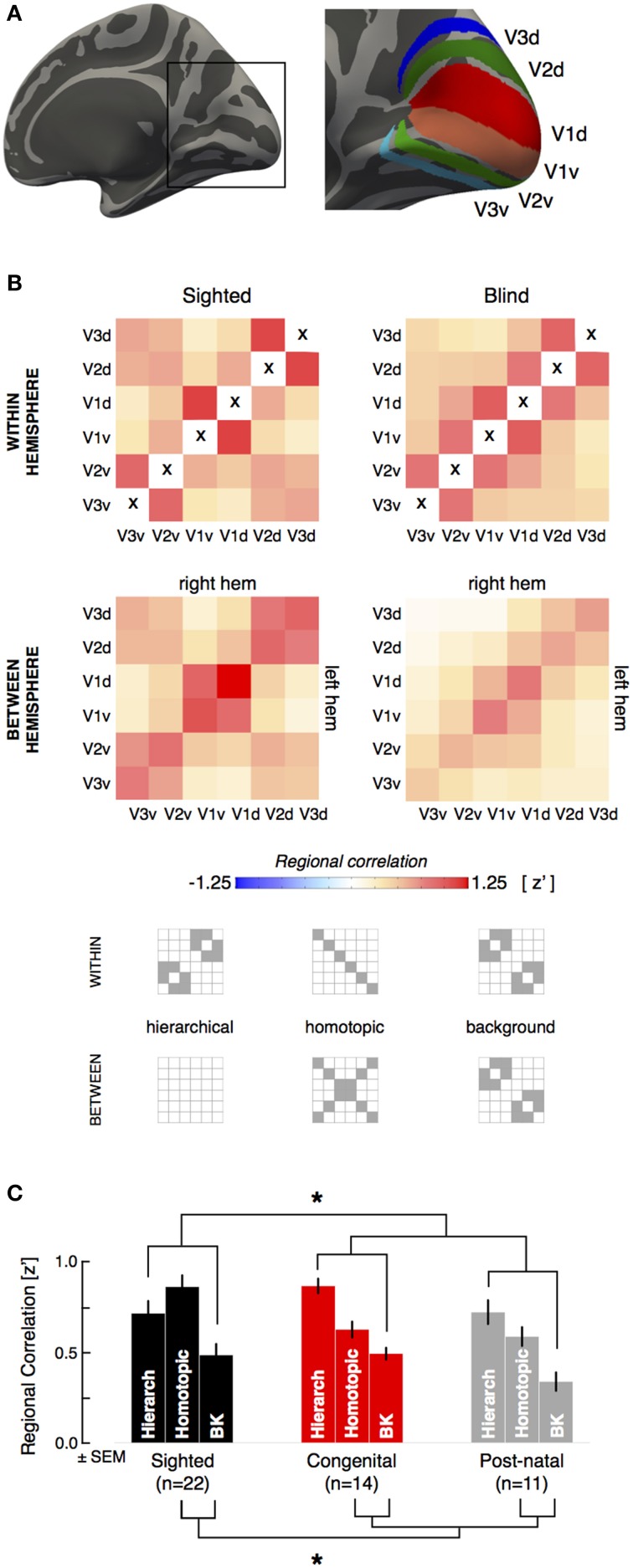
**Visual areas and whole-region correlation. (A)** The dorsal and ventral aspects of visual areas V1–V3 were identified by reference to cortical surface topology. There are six “quarter area” cortical representations in each brain hemisphere, corresponding to the dorsal and ventral aspects of visual areas V1–V3. Note borders between visual regions are not included in the ROI definitions. **(B)** The across subject, average correlation matrix is shown for within hemisphere and between hemisphere region relations, in sighted (*n* = 22) and blind (*n* = 25) subjects. Within hemisphere, the cells on the positive diagonal represents the correlation of a region with itself and are marked with an “x” as these values are undefined in our analysis. **(C)** Average hierarchical, homotopic, and background regional correlations for the sighted and blind groups. The lines and asterisk indicate significant (*p* < 0.05) statistical comparisons between groups (combining the congenital and postnatally blind).

Anatomical regions-of-interest (ROIs) corresponding to the dorsal and ventral halves of V1 through V3 (Figure 1A) were defined using the cortical surface template of retinotopy reported by Benson et al. (2014). Briefly, this template parcellates occipital cortex into V1, V2, and V3 regions and provides a retinotopic assignment (angle and eccentricity) for each vertex. The template was derived from retinotopic mapping data from 19 subjects that were aggregated within the fsaverage-sym cortical surface space. The aggregated retinotopic mapping data were fit with an algebraic model of retinotopic organization. The resulting template maps points on a standard cortical surface atlas (fsaverage-sym) to visual areas and their retinotopic assignment. The accuracy of prediction provided by the anatomical atlas for any one subject is comparable to the accuracy of a 20 min functional MRI scan.

All ROIs excluded the border between regions (defined as ±10° polar angle from the vertical or horizontal meridian border between visual areas). For each subject, the Pearson's correlation of the mean resting signal (defined as the average signal of all vertices comprising a given region) between each pair of regions was obtained within a given hemisphere. Each pair-wise visual quadrant correlation provided one cell of a resulting 6 × 6 correlation matrix. This within-hemisphere (ipsilateral) matrix is symmetric about the diagonal. Similarly, the Pearson's correlation of the mean resting signal between each pair of regions across hemispheres was obtained. Here, the resulting 6 × 6 correlation matrix is not necessarily symmetric, as all regions in the left hemisphere were compared to those in the right hemisphere. Following a Fisher's r-to-z transformation, one ipsilateral correlation matrix (an average of the right and left hemisphere correlation matrices), and one between-hemisphere correlation matrix was generated for each subject. Cells of the correlation matrices that corresponded to direct, “hierarchical” regional pairings and to indirect, “homotopic” regional pairings were averaged. Those cells which were not either hierarchical or homotopic were grouped as “background” pairings.

The lateral geniculate nucleus (LGN) was defined within standard volumetric (MNI) space using the Juelich Histological atlas (Eickhoff et al., 2005). The anatomical image from each subject was first registered to a template in MNI space using SPM8. The LGN region in MNI space was then projected back to the anatomical space for each subject via an inverse of the transformation matrix. The time series of all voxels within the LGN mask were averaged to obtain an average LGN signal for each subject.

### Visualizing fine-scale correlation: radial symmetry plot

Figure S3 provides a schematic description of the analysis approach. Analyses of the fine-scale correlation between regions were conducted following removal by least-squares regression of the mean time-series signal from each visual area. The visual area definitions supplied by cortical surface topology also contain polar angle and eccentricity assignments for each surface vertex (Benson et al., 2014). A single “seed” vertex was selected from a source visual area, and the correlation of this seed to each vertex in a target area was obtained between 1 and 15° eccentricity (corresponding to the region of greatest prediction accuracy of the cortical surface template; Benson et al., 2014). A given visual area could serve as both the source of the seed vertex and be the target. The set of correlation values were then placed in a polar plot, with position assigned by the polar angle and eccentricity of the vertex in the target area. Eccentricity values were expressed as the log_10_ of the ratio of the eccentricity of each target vertex to the eccentricity of the seed vertex, thus aligning all plots to a common relative eccentricity. This process was then repeated for every seed vertex in the source region, and the resulting set of plots were averaged to produce a radial symmetry plot. This approach closely follows that developed by Bao and Tjan (2009).

### Tests of fine-scale group differences

Each quadrant of each radial plot was averaged across angular values. These data were then fit for each quadrant, for each visual region pairing, for each subject with a Gaussian function for amplitude (z′) and width (log_10_ ratio of seed and target eccentricities). Fine-scale hierarchical and homotopic correlations for one blind subject (B01) was excluded due to poor model fit (*R*^2^ = 0.01, 0.15) of both the fine-scale hierarchical and homotopic correlations. A second blind subject (B25) similarly was excluded due to poor model fit of only the fine-scale homotopic correlations (*R*^2^ = 0.60). Fits were otherwise excellent (median adjusted *R*^2^ > 0.90 across individual fits). Group differences were then assessed using unpaired *t*-tests on individual fits.

## Results

The imaging data from each subject were aligned to a hemisphere-symmetric, cortical-surface template that defines retinotopy and visual areas V1 through V3 by sulcal topology (Figure 1A; Benson et al., 2012, 2014). The visual areas were divided into their dorsal and ventral halves (each of which represents a quarter of the visual field; i.e., quarter-areas). Data from within 10° polar angle of the borders between visual areas were excluded to avoid correlations between visual areas attributable to local image smoothness or callosal connections along the vertical meridian (Clarke and Miklossy, 1990). We examined the correlation of the blood oxygenation level dependent (BOLD) signal between these regions within and across the hemispheres for sighted and blind participants.

### Blindness enhances hierarchical and decreases homotopic correlations within visual cortex

Prior studies have shown that blindness decreases between-hemisphere correlations in the occipital lobe (Liu et al., 2007; Yu et al., 2008; Watkins et al., 2012; Butt et al., 2013; Burton et al., 2014). We obtained the correlation between the left and right hemisphere for the average fMRI signal within all of V1–V3 cortex for each subject. Consistent with prior reports, the entire between-hemisphere correlation of the visual cortex was reduced in the blind as compared to the sighted (sighted vs. blind: two-tail *t*[45 *df*] = 5.0, *p* = 0.0000093). We then examined how the relative strength of connections within and between hemispheres was related to position in the visual hierarchy.

The set of correlations from each pairing of visual area quadrants within a hemisphere, and across hemispheres, can be expressed in a matrix. Figure 1B presents these visual quadrant correlation matrices for the blind and sighted subjects (see Figure S1 for the same matrices for the congenital and postnatally blind subgroups). Structure can be seen within each matrix corresponding to increased correlations between visual areas with a hierarchical relationship, and increased correlations between the spatially distributed quarter fields of a given visual area (e.g., V2d and V2v in the left and right hemisphere). These indirect correlations have also been termed “homotopic” (Jo et al., 2012; Watkins et al., 2012), and with the exception of sparse monosynaptic connections adjacent to the vertical meridian (Clarke and Miklossy, 1990; Jeffs et al., 2009), these regions do not have direct neuroanatomical coupling. Control studies conducted using a water phantom show that this correlation structure is not the product of the MR imaging itself (see Figure S2). Some pairings do not reflect either hierarchical or homotopic relationships. We consider the correlation between these cortical patches to reflect the “background” correlation of unrelated areas of visual cortex. The strength of correlation of “background” pairings did not differ between the blind and sighted groups (whole group: *t*[45 df] = −0.9, *p* = 0.40; age-matched: *t*[28 *df*] = −0.3, *p* = 0.78). We consider the strength of hierarchical and homotopic correlations relative to this background level of correlation.

We compared the blind and sighted groups in the average correlation for hierarchical and homotopic regional pairings (Figure 1C). Across the populations, the blind subjects had a greater correlation between cortical visual areas with a hierarchical relationship relative to the background (*t*[45 *df*] = 3.7, *p* = 0.00060). In contrast, the blind subjects had significantly reduced correlation between the homotopic pairings of distributed quarter-fields of the same visual area relative to background pairings (*t*[45 *df*] = −3.7, *p* = 0.00057]). The interaction between hierarchical and homotopic pairings was also significant (*t*[45 *df*] = 4.6, *p* = 0.000031). We confirmed that the same pattern of results were observed when conducted in age matched sub-groups (sighted vs. blind, hierarchical: *t*[28 *df*] = 2.9, *p* = 0.0066; homotopic: *t*[28 *df*] = −3.2, *p* = 0.0038).

The set of regions with homotopic pairing includes both diagonal and horizontal relationships (i.e., those pairings which reflect symmetry about both meridia, and those pairings which reflect symmetry only across the vertical meridian). Group differences between the sighted and blind were separately significant for both the diagonal (two-tail *t*[45 *df*]= −4.3, *p* = 0.00010; means: [0.42, 0.74], diff = −0.31) and horizontal components (two-tail t[45 *df*]= −3.5, *p* = 0.0012; means: [0.7, 3 1], diff = −0.28).

The congenital and postnatally blind groups did not differ from each other either in hierarchical or homotopic correlations (congenital vs. postnatal, hierarchical: *t*[23 *df*] = 1.9, *p* = 0.075; means: [0.87, 0.72]; homotopic: *t*[23 *df*] = 0.54, *p* = 0.60; means: [0.63, 0.59]), suggesting that these effects are not related to the early developmental effects. Figure S1 provides the whole-region correlation matrices for the congenital and postnatally blind subgroups. Table S2 presents all the main results for the study, divided by separate visual area pairings.

**Figure 2:**
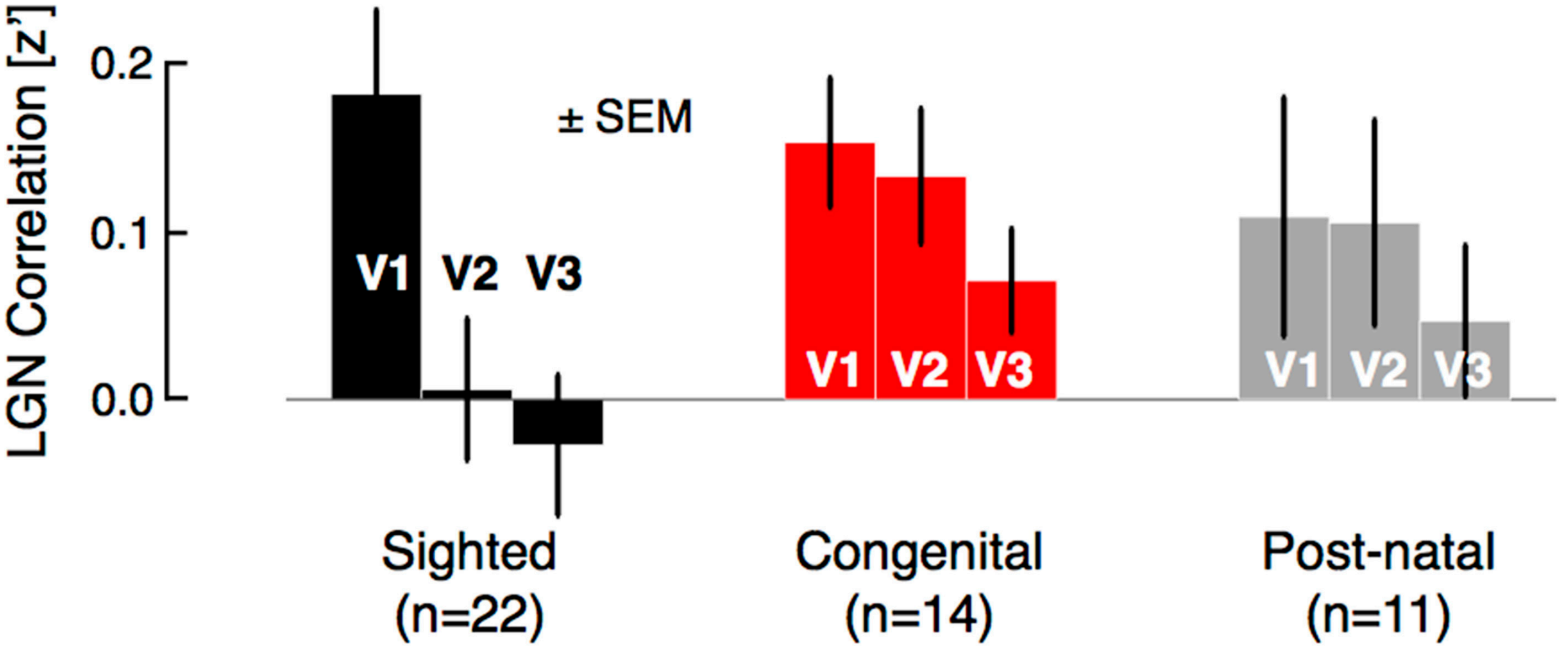
**Group comparisons of regional-level correlation between LGN and visual areas V1, V2, and V3**. For each subject, the time-series of voxels corresponding to the LGN were identified and averaged, then correlated with the average time-series of areas V1, V2, and V3. In the sighted group, the mean LGN signal is strongly correlated with area V1 signal as compared to later areas in visual hierarchy. No such dramatic drop in correlation was observed in the blind population, which was further divided into congenital and post-natal subgroups.

We then examined an additional form of hierarchical correlation within the visual system by identifying the lateral geniculate nucleus (LGN) in volumetric space for each subject and obtaining the correlation between the mean BOLD fMRI signal in the LGN and ipsilateral visual areas V1, V2, and V3 (Figure 2). In both the sighted and the blind, there was correlation between the LGN and area V1, and this did not differ between the groups (*t*[45 *df*] = 0.8, *p* = 0.44). A positive correlation with the LGN persisted for the blind within area V2 and nearly within V3 (sighted vs. blind, V2: [45 *df*] = 2.1, *p* = 0.040; V3: *t*[45 *df*] = 1.8, *p* = 0.075). There was no difference between the congenital and postnatally blind (all *p* > 0.5). This pattern of results suggest that both the blind and the sighted have a similar amplitude of shared signal between V1 and LGN, but that in the blind this shared signal remains minimally altered along the visual hierarchy.

Could the differences between the blind and sighted groups be a consequence of differences in head motion? Head motion enhances correlations locally and between symmetric hemispheric locations, while decreasing long range correlation between regions (Power et al., 2012; Van Dijk et al., 2012). These reported confounding effects do not map neatly onto the measurements we have made, as our “homotopic” correlation (for example) is a mix of both hemisphere symmetric and within hemisphere (dorsal/ventral) measures. Nonetheless, we tested for group differences in head motion by calculating the mean Euclidean displacement of the head during the resting-state scan for each subject (Van Dijk et al., 2012). The small difference between the groups was not significant (blind group displacement = 0.09 mm ± 0.055 *SD*, sighted group displacement = 0.07 mm ± 0.043 *SD*; *t*[45 *df*] = 1.4, *p* = 0.17). Further there was no significant correlation between individual subject head motion measures and the measurement of hierarchical or homotopic correlation across subjects, including their interaction (all *p*-values > 0.5).

### Fine-scale correlation between visual areas is organized along the eccentricity dimension for both the sighted and blind

**Figure 3:**
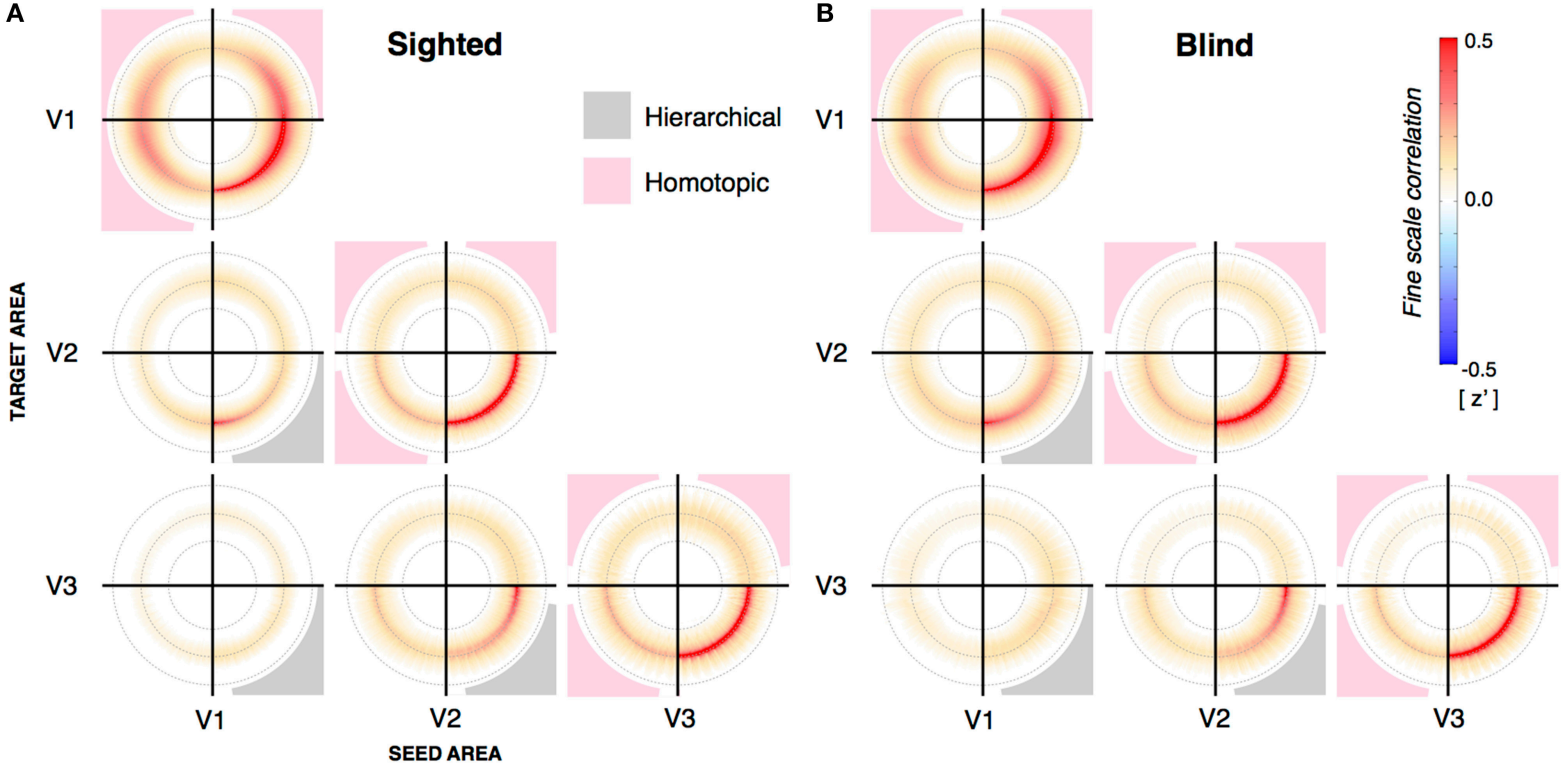
**Radial symmetry plots from sighted and blind subjects. (A)** Average radial symmetry plots from sighted subjects (*n* = 22) between V1, V2, and V3. Each subplot represents the correlation between a source and target visual area. This may be the same area (such as V1 to V1), or between different areas aligned by shared eccentricity assignment. The quadrants reflecting direct, hierarchical correlation are shaded in gray. When a given visual area is compared to itself, this same lower right quadrant contains an area of elevated correlations resulting from the physical adjacency of points in the imaging volume. **(B)** Average radial symmetry plots from blind subjects (*n* = 25) between V1, V2, and V3.

The cortico-cortico connective fields in visual areas are on the order of several millimeters (Stepniewska and Kaas, 1996; Van den Bergh et al., 2010). We considered the possibility that differences between the congenital and postnatally blind groups might be present at this smaller scale, and not revealed in the synchronous, aggregate behavior of cortical regions. We characterized the eccentricity aligned structure of fine-scale correlations between visual areas (Yeo et al., 2011; Jo et al., 2012; Butt et al., 2013; Raemaekers et al., 2013; Buckner and Yeo, 2014) in a radial symmetry plot (Bao and Tjan (2009); Figure S3). These plots capture the change in signal correlation between cortical points as a function of change in log relative eccentricity. Figure 3 presents the average radial symmetry plots for the sighted and blind subjects, for each pairing of visual area. The prominent “ring” in each plot indicates that there is an elevated correlation between cortical points that share similar eccentricity assignments, whether the two points are in the same visual area, hierarchically associated visual areas, or spatially separated quadrants of the same visual area. For plots within a visual area (e.g., V1 → V1), the lower right quadrant contains locally elevated values. These correlation values are derived from points that are physically adjacent within the imaging volume, and thus have elevated correlation values due to shared signal from imaging and digital smoothing effects (Butt et al., 2013). This is supported by control studies using a water phantom that replicate the elevated values at this location. (see Figure S4). Importantly, however, these non-physiologic sources of signal correlation make a minimal contribution to the other quadrants of the radial symmetry plots.

### Blindness alters the relative amplitude of fine-scale correlations

**Figure 4:**
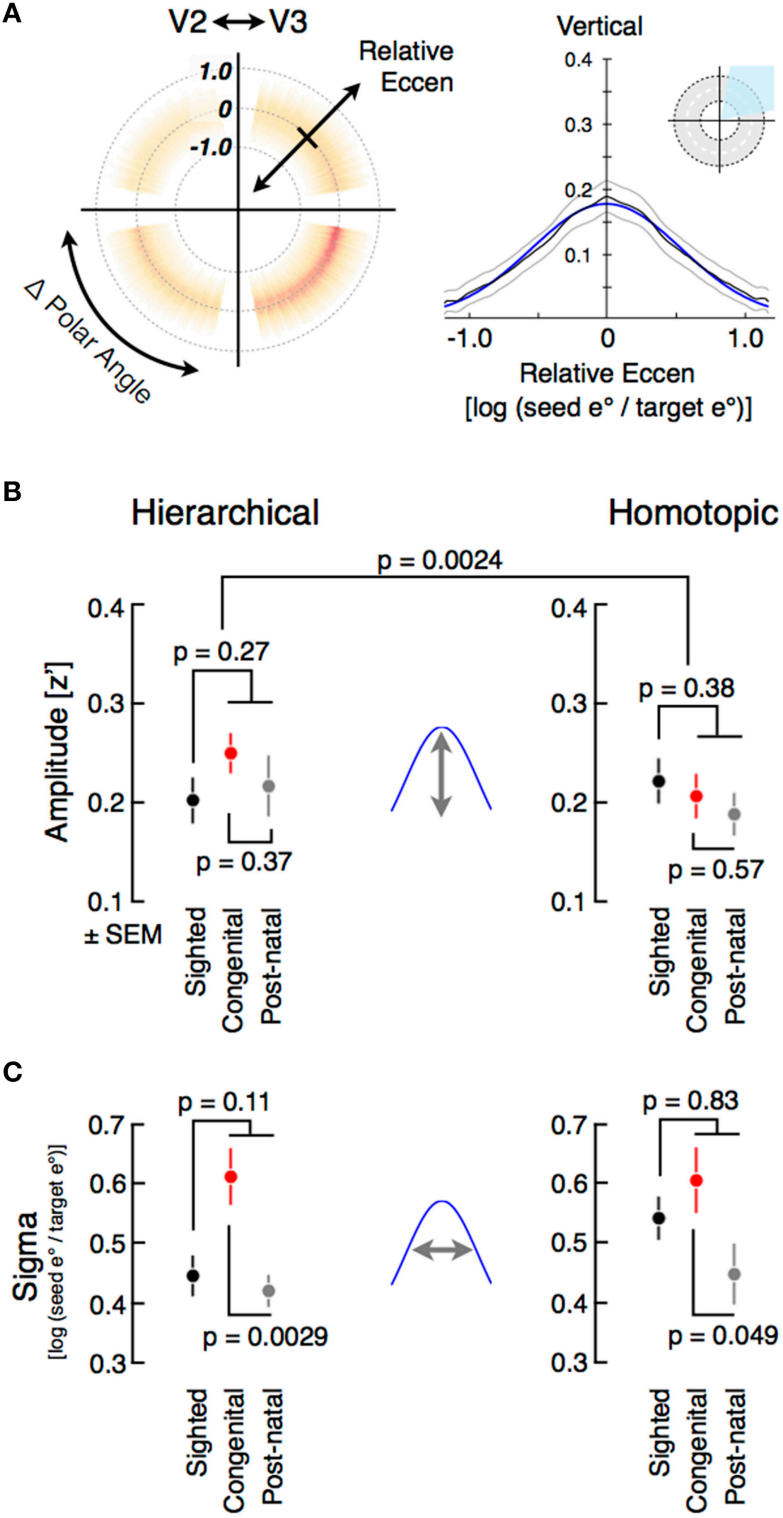
**Modeling fine-scale correlation and group comparisons of fine-scale hierarchical (direct) and homotopic (indirect) correlation amplitude and width**. **(A)** The average radial symmetry plot from sighted subjects (*n* = 22) for V2 source points and V3 target points. Each quadrant of the plot indicates the correlation of cortical points with symmetry across the vertical or horizontal meridian (or both), or hierarchical relations within a visual quadrant. As with the whole-region ROIs, cortical points at the borders between visual areas (defined as within 10° of the meridia) were excluded. Fine-scale correlation is well modeled by a Gaussian function of log eccentricity ratio (adjusted *R*^2^ > 0.90), the parameters of which are amplitude and width (sigma). **(B)** For each subject, the correlation in the quadrants reflecting hierarchical and homotopic connections were averaged prior to model fitting. Individual amplitude fits were obtained, and then averaged across all subjects within a group, including further subdividing the blind group into the congenital and post-natal subgroups. **(C)** Average correlation widths from sighted and blind subjects.

We extracted from radial symmetry plots a measure of the amplitude and width of eccentricity-organized correlations. The data from each quadrant of each plot was collapsed along polar angle, excluding those cortical points that fell within 10° of the borders of visual areas. Points along the visual area borders were excluded to prevent local image smoothness, unrelated to neural coupling, from contributing to the measured correlations (Figure S4A). A plot of correlation as a function of change in log relative eccentricity (Figure 4A right) demonstrates that the relationship is well characterized by a Gaussian function. The amplitude and width of a Gaussian fit to the eccentricity function was obtained from each subject for each quadrant of the radial symmetry plots. These measures of fine-scale correlation structure were separately aggregated for pairs of cortical areas with a hierarchical or homotopic relationship.

The blind population had a larger amplitude of fine-scale correlations between eccentricity-coupled positions with a hierarchical relationship, and a smaller amplitude of correlations between the spatially distributed quarter fields of a given visual area (Figure 4B). While these differences themselves were not significant (hierarchical: two-tail *t*[44 *df*] = 1.1, *p* = 0.27; means: [0.23, 0.2]; homotopic: two-tail *t*[43 *df*] = −0.9, *p* = 0.38; means: [0.2, 0.22], the interaction of group and form of correlation was significant (two-tail *t*[43 *df*] = 3.2, *p* = 0.0024), as found previously at the regional level. This interaction was present as well in age-matched sub-groups of blind and sighted subjects (two-tail *t*[27 *df*] = 3.2, *p* = 0.0035).

Following the same logic applied to the analysis of regional correlations, we asked if the effect of blindness upon the amplitude of fine scale correlations differed between the congenitally and postnatally blind. Again, as was seen for regional correlations, there was no significant effect of age of onset of blindness upon the amplitude of fine-scale correlations. Therefore, these results suggest that the amplitude of both hierarchical and homotopic correlations at both the regional and fine-scale level is the result of sustained removal of visual input, as opposed to possible developmental effects of blindness.

### The width of fine-scale correlations is altered by congenital blindness

Early visual deprivation is associated with broader cortico-cortico projection zones between visual areas in animal models (Fish et al., 1991; Olavarría et al., 2008; Zufferey et al., 1999). Consistent with this, we found that the width of eccentricity organized, fine-scale correlations between hierarchical visual areas was broader for the congenitally blind group compared to the postnatally blind group (Figure 4C; *t*[22 *df*] = 3.4, *p* = 0.0029; means: [0.61, 0.42]). A similar difference was found between homotopic visual quadrants (*t*[21 *df*] = 2.1, *p* = 0.049; means: [0.61, 0.45]). Interestingly, this effect of blindness upon the spatial extent of fine-scale correlation was mainly present for the congenitally blind group, as the correlation width observed for the postnatally blind group was similar to that found for the sighted for both the hierarchical and homotopic fine-scale correlations. This difference is visually apparent in the radial symmetry correlation plots for the congenital and postnatally blind groups (Figure S5). When considered as a combined group, no significant difference was observed between the blind and sighted subjects (hierarchic: *t*[44 *df*] = 1.6, *p* = 0.111; means: [0.52, 0.45]; homotopic: two-tail *t*[43 *df*] = −0.2, *p* = 0.83; means: [0.53, 0.54], *d*iff = −0.012). We interpret this alteration of spontaneous correlation structure in the blind as reflecting primarily early developmental changes, perhaps related to altered cortical maturation.

We considered the possibility that the difference in correlation width between the blind and sighted groups is a result of differences in surface area of visual cortex. If it were the case that fine-scale correlations have the same width across groups in millimeters of cortical distance, then a reduction in the relative size of the visual cortex in blind subjects could be mistaken for a broadening of correlation width. A reduction in the surface area of V1 (relative to the surface area of the entire brain) has been described previously in the blind compared to sighted controls (Park et al., 2009), and we observe a similar effect in our data (two-tail *t*[45 *df*]= −5.1, *p* = 0.000006; means: [0.024, 0.029]). However, there was no difference in surface area between the sighted and blind groups for areas V2 and V3 (two-tail *t*[45 *df*]= −0.6, *p* = 0.53; means: [0.044, 0.045]; see Table S3). Further, the correlation widths (including the hierarchical/homotopic interaction terms) measured for each participant were not themselves correlated across subjects with the relative surface area of V1, V2, V3, or V2+V3 (all *p*-values > 0.05 for both Spearman and Pearson correlation tests). Therefore, differences in fine-scale correlation width do not appear to be explained by differences in visual cortex surface area.

Finally, our findings depend upon the ability to register individual brains to a common cortical surface template (Benson et al., 2014). Systematically different cortical surface topology in the blind population could confound our observed group differences. We tested this by obtaining for each subject the mean and standard deviation of the log Jacobian areal warp values (Fischl et al., 2001) calculated for each surface vertex within V1, V2, and V3. This value indexes the degree to which the cortical surface of a given subject required warping to fit the common surface atlas. After accounting for group differences in mean surface area (which contributes to the Jacobian measure), there was no difference in either mean or standard deviation of the Jacobian between the groups for V1, V2, or V3 (all *p*-values > 0.5).

## Discussion

In the absence of extensive monosynaptic connections, the synchronization of signals between the quadrants of a visual area—separated vertically and across the hemispheres—must be achieved indirectly by shared input signals. In contrast, signals intrinsic to a patch of cortex can propagate by a monosynaptic route directly along the visual hierarchy. We find that the relative correlation strength of indirect homotopic and potentially hierarchical signals is altered by vision loss. Moreover, the timing of blindness impacts the fine-scale structure of correlations in a manner suggestive of altered cortical maturation.

Previous studies examining ipsilateral (Liu et al., 2007; Burton et al., 2014) and inter-hemispheric correlation (Liu et al., 2007; Yu et al., 2008; Watkins et al., 2012; Burton et al., 2014) at the level of entire cortical regions show that blindness decreases the spontaneous correlation between the separated quadrants of a visual area. The current study replicates this result, and further shows that this decrease in homotopically related cortical areas is accompanied by a relative increase in the correlation between hierarchically related visual areas. This effect is found to extend as well to the correlations that relate the LGN to the cortical visual areas.

In addition to an analysis of correlation structure at the level of entire regions, the use of an anatomical template of retinotopy (Benson et al., 2014) allowed us to examine the fine-scale structure of correlations between cortical points with matched eccentricity values. In a prior study of these data (Butt et al., 2013), we examined these fine-scale correlations in the sighted and blind groups, but confined our analyses to area V1. As in the current study, there was no difference in between-hemisphere amplitude of fine-scale correlations between the blind and the sighted after accounting for group differences in the correlation of entire visual regions. With the extension of our anatomical template of retinotopy to extra-striate cortex, we examine correlation structure across visual areas. With this wider view, we find here that the same relative enhancement of correlation amplitude for hierarchically related cortical points is found on the fine scale level, relative to homotopically related locations.

The extension of our work to extrastriate cortex has also allowed us to re-examine the width of cortico-cortico correlation structure within the sighted and the blind. In our prior study confined to area V1, we found a wider spread of fine-scale correlations in the blind as compared to the sighted, but could not distinguish this effect from a reduction in the surface area of V1 found in blindness. In the current study, the ability to examine fine-scale correlations within extra-striate visual areas—which do not differ in surface area between the two groups—allowed us to confirm that there is in fact a widening of cortico-cortico correlations, although this effect was present only in the congenitally blind.

Our study makes use of an atlas of cortical surface topology to assign visual areas and retinotopic organization to cortical points without the need for functional retinotopic mapping. This enables a comparison of blind and sighted subjects in a common framework. A prerequisite of such a comparison is that both groups of subjects are represented with equal fidelity within the anatomical space. Accordingly, we confirmed that there is no difference between the blind and sighted groups in the quality of the registration of brain anatomy from individual subjects to the cortical template. This may not be the case for other populations of subjects. For example, people with cortical lesions or malformations will have a systematically different registration of brain anatomy to the template space as compared to control populations. This difference would then complicate the interpretation of differences in functional organization within the template space.

**Figure 5:**
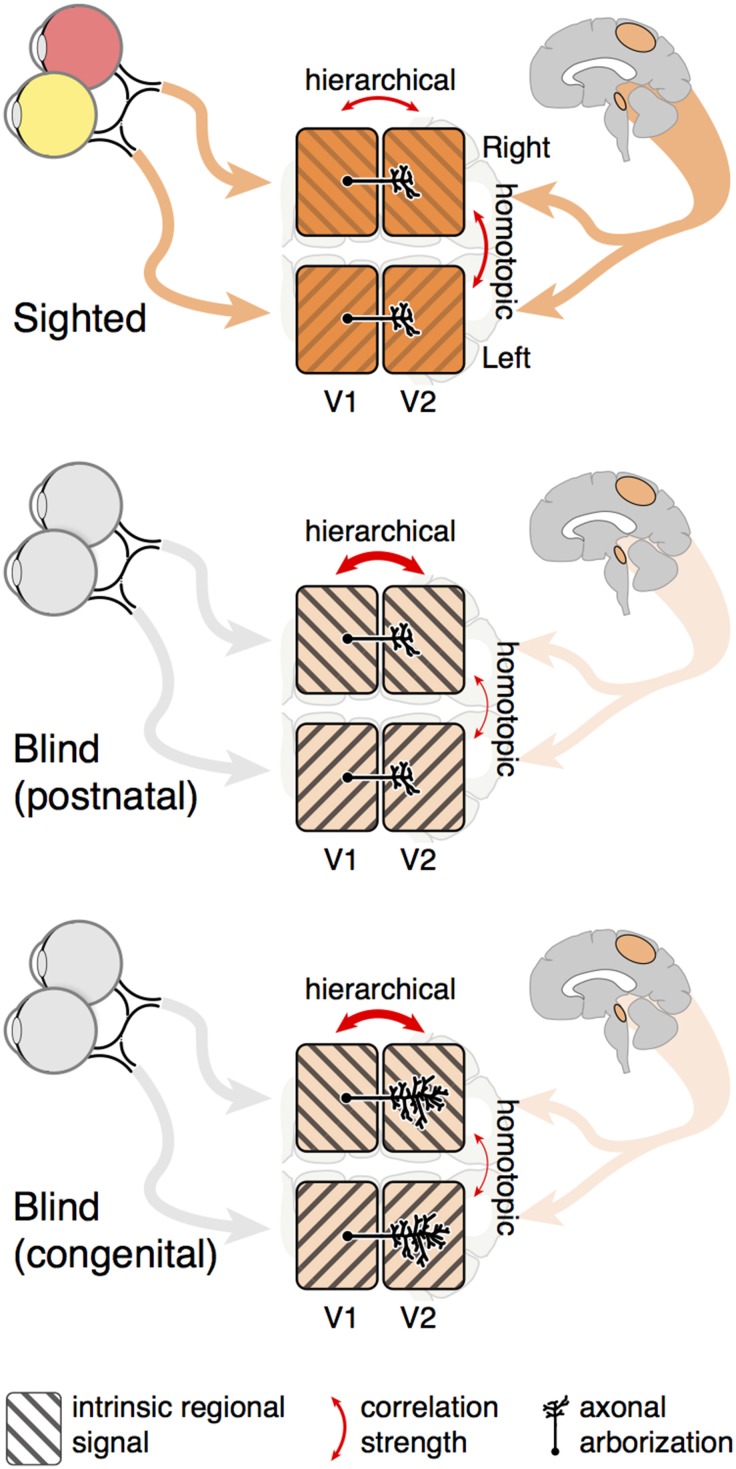
**Summary model**. The correlation of spontaneous signals between cortical visual areas is influenced by both shared input signals and intrinsic neural activity. Blindness reduces shared, between-hemisphere input to visual areas, from both retinal and higher cortical sources. The signals intrinsic to hierarchical, connected visual areas therefore constitute a greater proportion of spontaneous neural activity, enhancing the relative strength of hierarchical correlation measures as compared to indirect. Congenital blindness has the additional effect of broadening between-visual-area axonal arborization and thus measured cortico-cortico receptive fields.

Figure 5 presents a simple model that accounts for our findings. In sighted individuals, the spatially separated hemifields of area V1 receive a common input signal from bottom-up and top-down sources. Bottom-up input could result from time-varying, spatially structured retinal ganglion cell activity (Wong, 1999) from each eye, mixed at the level of the LGN. Higher-order cortex, containing receptive fields that cross the vertical and horizontal meridia, can also provide top-down synchronization of the visual cortex quadrants via reciprocal connections.

In addition to shared input, each separated patch of area V1 has its own intrinsic, spontaneous signal, perhaps emerging from coherent neural oscillations within or between areas (Usrey and Reid, 1999). A mixture of the shared retinal input and the intrinsic, quadrant specific V1 signal is then propagated to higher visual areas via cortico-cortico connections. Intrinsic signals are a combination of oscillatory activity across multiple frequency bands, and have previously been shown to be altered in congenital blindness (Hawellek et al., 2013). Therefore, blindness reduces synchronizing retinal input while simultaneously increasing the relative contribution of intrinsic regional signal to functional correlation. The consequence for functional MRI measures is an increase in the correlation of directly connected, hierarchical regions as compared to spatially separated quadrants of a particular visual area (Figures 1C, 4B). This effect is present at both the regional and fine scale, and is a common effect of blindness, regardless of the timing of vision loss. When the width of cortico-cortical connections are examined, we find that congenital blindness is additionally associated with a widening of hierarchical correlations (Figure 4C). This is seen for both hierarchical and homotopic correlation measures, as the monosynaptic, hierarchical connections carry both the shared input and local, intrinsic regional variations.

Between-hemisphere synchronization must in part be supported by a top-down signal, as it persists in anophthalmic patients (Watkins et al., 2012). Computational models suggest that a network of reciprocal connections, more than a shared direct input, is responsible (Gollo et al., 2014). As an example of altered, shared signal, blindness has been found to differentially alter the correlation of the left and right visual cortex with frontal language areas (Bedny et al., 2011), and the size of this change is related to the degree of de-synchronization of left and right V1 signals (Butt et al., 2013). In the now deafferented visual cortex, lateralized processing of non-visual information such as episodic recall and attention may predominate (Burton et al., 2014). Interestingly, sectioning of the corpus callosum does not abolish correlation between the hemispheres (Uddin et al., 2008), leaving sub-cortical connections (e.g., via a collicular-thalamic route) as a possibility (Savazzi and Marzi, 2004; Savazzi et al., 2007).

We measured the width of fine-scale correlations between visual areas. The spatial scale of this correlation structure (a log eccen ratio of ~0.45 corresponds to approximately 3–8° visual angle for eccentricities less than 15°) is commensurate with the ~8° eccentricity suppressive surrounds observed in V2 neurons in the macaque (Van den Bergh et al., 2010). We found a broader spread of fine-scale correlations in the congenitally blind. This could reflect the altered cortical maturation process that has been observed in animal models. Axonal projections with rudimentary retinotopic organization are established between hierarchical visual areas prenatally (Coogan and Van Essen, 1996). Shortly after birth, visual input guides these axonal projections to their retinotopically linked targets (Innocenti and Price, 2005; Ruthazer et al., 2010; Baldwin et al., 2012). Disruption of vision during this peri-natal period causes the axon terminals to reach more broadly within the target visual cortex (Bock and Olavarría, 2011). The presence of a neonatal sensitive period is further supported by research in higher order visual areas, such as MT/MST, in which congenital blindness infers differential connectivity to lateral prefrontal areas compared to post-natal blindness (Bedny et al., 2010).

The eccentricity-aligned organization of spontaneous signals in visual cortex has been described previously (Bao and Tjan, 2009; Yeo et al., 2011; Raemaekers et al., 2013; Buckner and Yeo, 2014). In previous work, we have shown that this signal is aligned with the eccentricity axis of cortex but does not reflect eccentricity representation *per se* (Butt et al., 2013). In our data we do not observe a signal component related to the orthogonal dimension of polar angle, although others have (Heinzle et al., 2011). Like others (Cohen et al., 2008; Raemaekers et al., 2013; Wig et al., 2014; Buckner and Yeo, 2014), we do find a signal component in the sighted that distinguishes striate from extra-striate cortex, and which can be explained by the presence of LGN signal within area V1 (Figure 2). As there are no monosynaptic connections that would result in a distribution of neural signal across polar angle, this feature of the data presumably arises from network-level properties of spontaneous activity to create organization along the eccentricity dimension.

Our work uses variations in visual experience to delineate the separate components of spontaneous neural activity within visual cortex. Post-natal visual experience sustains network-level neural activity in darkness and synchronizes firing between spatially distributed cortical visual areas. Congenital blindness disrupts normal cortical maturation, broadening neural signals between hierarchical visual areas. An application of this finding is to the selection and study of patients undergoing treatment for forms of early blindness. Despite arriving at an equally severe destination of visual impairment, different clinical trajectories of vision loss can result in more or less developmental alteration of visual cortex. We can separably assess the developmental and ongoing effects of blindness upon the visual cortex, with each having potentially different prognostic value for functional visual recovery. Measurement of spontaneous neural signals is therefore a means to probe the properties of visual cortex without vision.

## Author contributions

Omar H. Butt and Geoffrey K. Aguirre designed research; Omar H. Butt performed research; Omar H. Butt, Noah C. Benson, Ritobrato Datta analyzed data; Omar H. Butt and Geoffrey K. Aguirre wrote the paper.

### Conflict of interest statement

The authors declare that the research was conducted in the absence of any commercial or financial relationships that could be construed as a potential conflict of interest.
