# Supplementary material for: Hierarchical and homotopic correlations of spontaneous neural activity within the visual cortex of the sighted and blind
Source: Front Hum Neurosci. 2015 Feb 10;9:25. doi: 10.3389/fnhum.2015.00025 (PMC4322716; doi:10.3389/fnhum.2015.00025)
Supplement: Supplementary file 3 [file Table3.PDF]

Table S3. Surface Area (%Total Surf ± SEM)

Region	Sighted Group	Blind Group
V1	2.9 (±0.074)	2.4 (±0.071)
V2	2.5 (±0.029)	2.4 (±0.033)
V3	2.0 (±0.028)	2.0 (±0.022)

Table S3 l Group comparison in surface area. For each subject, the cortical surface area for each visual area was obtained and scaled by total brain surface area. Only the relative surface area of V1 was significantly smaller in the blind group as compared with the sighted group.
