# Supplementary material for: Hierarchical and homotopic correlations of spontaneous neural activity within the visual cortex of the sighted and blind
Source: Front Hum Neurosci. 2015 Feb 10;9:25. doi: 10.3389/fnhum.2015.00025 (PMC4322716; doi:10.3389/fnhum.2015.00025)
Supplement: Supplementary file 4 [file Image1.PDF]

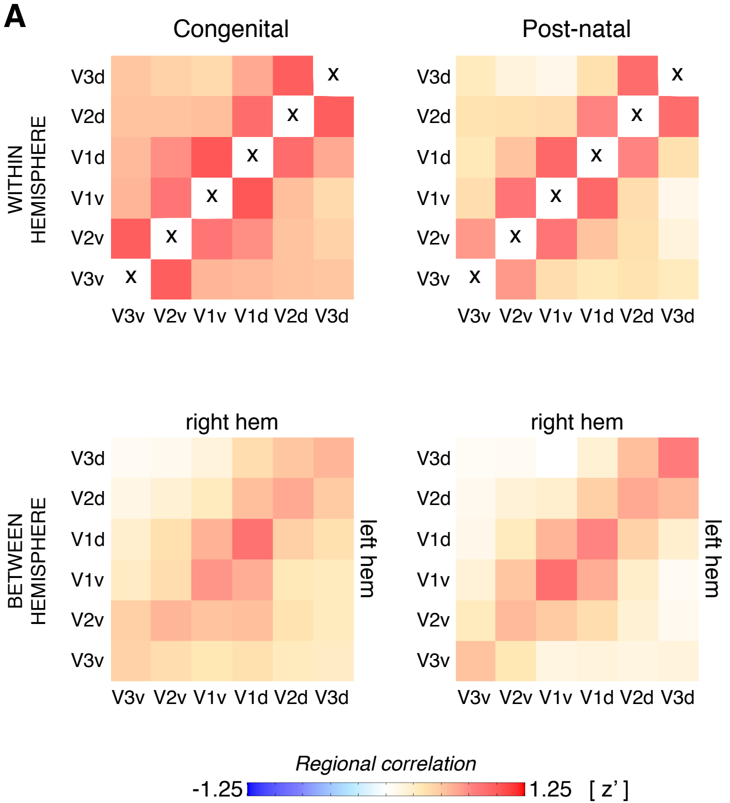

FIGURE S1 | Whole-region correlation matrices for the blind subjects, divided into the congenital (n=14) and postnatally (n=11) blind groups. The across subject, average correlation matrix is shown for within hemisphere and between hemisphere region relations. Within hemisphere, the diagonal represents the correlation of a region with itself and is therefore not shown. The cells that represent hierarchical relationships between quarter areas are outlined.
