# Supplementary material for: Hierarchical and homotopic correlations of spontaneous neural activity within the visual cortex of the sighted and blind
Source: Front Hum Neurosci. 2015 Feb 10;9:25. doi: 10.3389/fnhum.2015.00025 (PMC4322716; doi:10.3389/fnhum.2015.00025)
Supplement: Supplementary file 5 [file Image2.PDF]

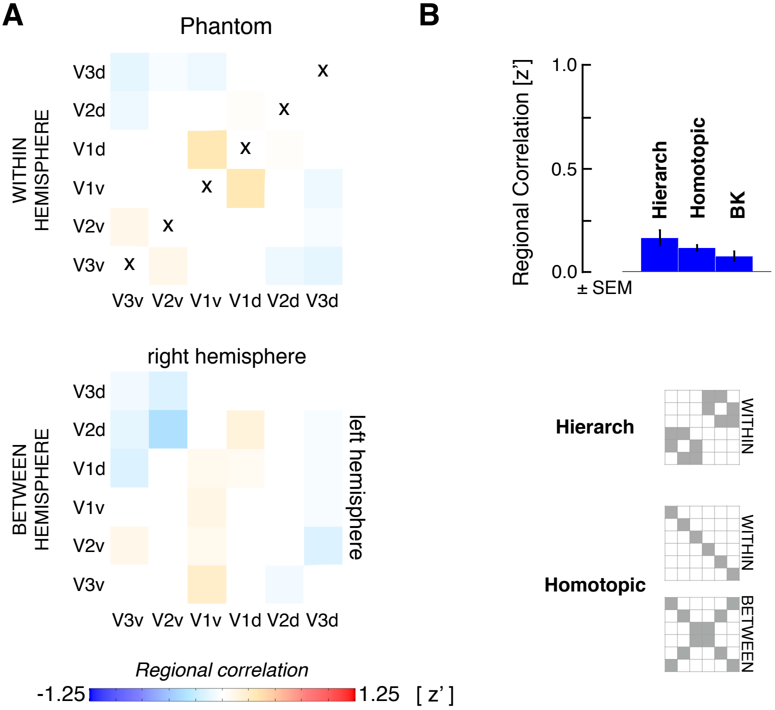

FIGURE S2 I Whole-region correlation matrices derived from phantom data. (A) Within and between hemisphere correlation matrices. Raw volumetric time-series data from 8 random subjects were replaced with data obtained from a water phantom scanned under otherwise identical conditions. Similar to Fig. 2A, the resulting correlation matrices for phantom data were then averaged following a Fisher's r-to-z transformation and the subtraction of the average correlation of the matrix from each cell. **(B)** Group comparisons of hierarchical (direct) and homotopic (indirect) regional correlations. The average of all cells reflecting hierarchical correlation, top, were plotted for the phantom-substituted data. A small, non-zero correlation was observed for hierarchical regional correlation within the water phantom, likely related to the physical adjacency of hierarchically related visual areas (e.g., V2d, V3d) projected into the space of the imaging volume. In contrast (bottom) no correlation above baseline was observed in homotopic correlation.
