# Supplementary material for: Hierarchical and homotopic correlations of spontaneous neural activity within the visual cortex of the sighted and blind
Source: Front Hum Neurosci. 2015 Feb 10;9:25. doi: 10.3389/fnhum.2015.00025 (PMC4322716; doi:10.3389/fnhum.2015.00025)
Supplement: Supplementary file 6 [file Image3.PDF]

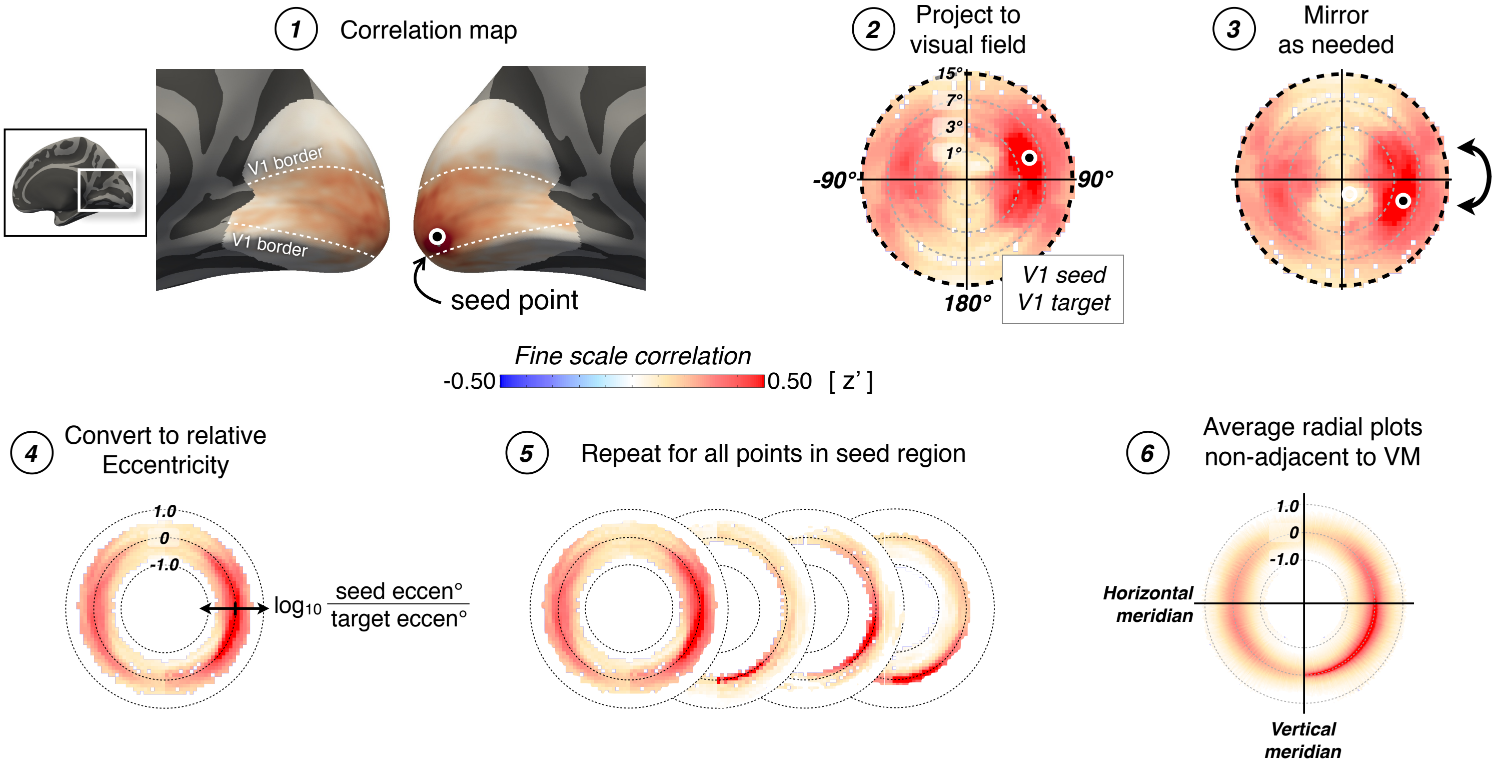

FIGURE S3 | Radial symmetry plots express the organization of fine-scale cortical correlation projected to the visual field (derived from Bao & Tjan 2009). (1) A seed point is selected in the source visual area (e.g., area V1) and a map of correlation between that seed point and the remainder of visual cortex is created. **(2)** The correlation map for the target visual area (also V1 in this example) is projected to the visual field. **(3)** The plot is mirrored as needed to place the seed point in the lower right quadrant. **(4)** All eccentricity values in the plot are divided by the eccentricity of the seed point, then the log10 of the ratio is obtained. This process is repeated for all seed points in a source region **(5)** and the average of all resulting plots obtained **(6)**.
