# Supplementary material for: Hierarchical and homotopic correlations of spontaneous neural activity within the visual cortex of the sighted and blind
Source: Front Hum Neurosci. 2015 Feb 10;9:25. doi: 10.3389/fnhum.2015.00025 (PMC4322716; doi:10.3389/fnhum.2015.00025)
Supplement: Supplementary file 7 [file Image4.PDF]

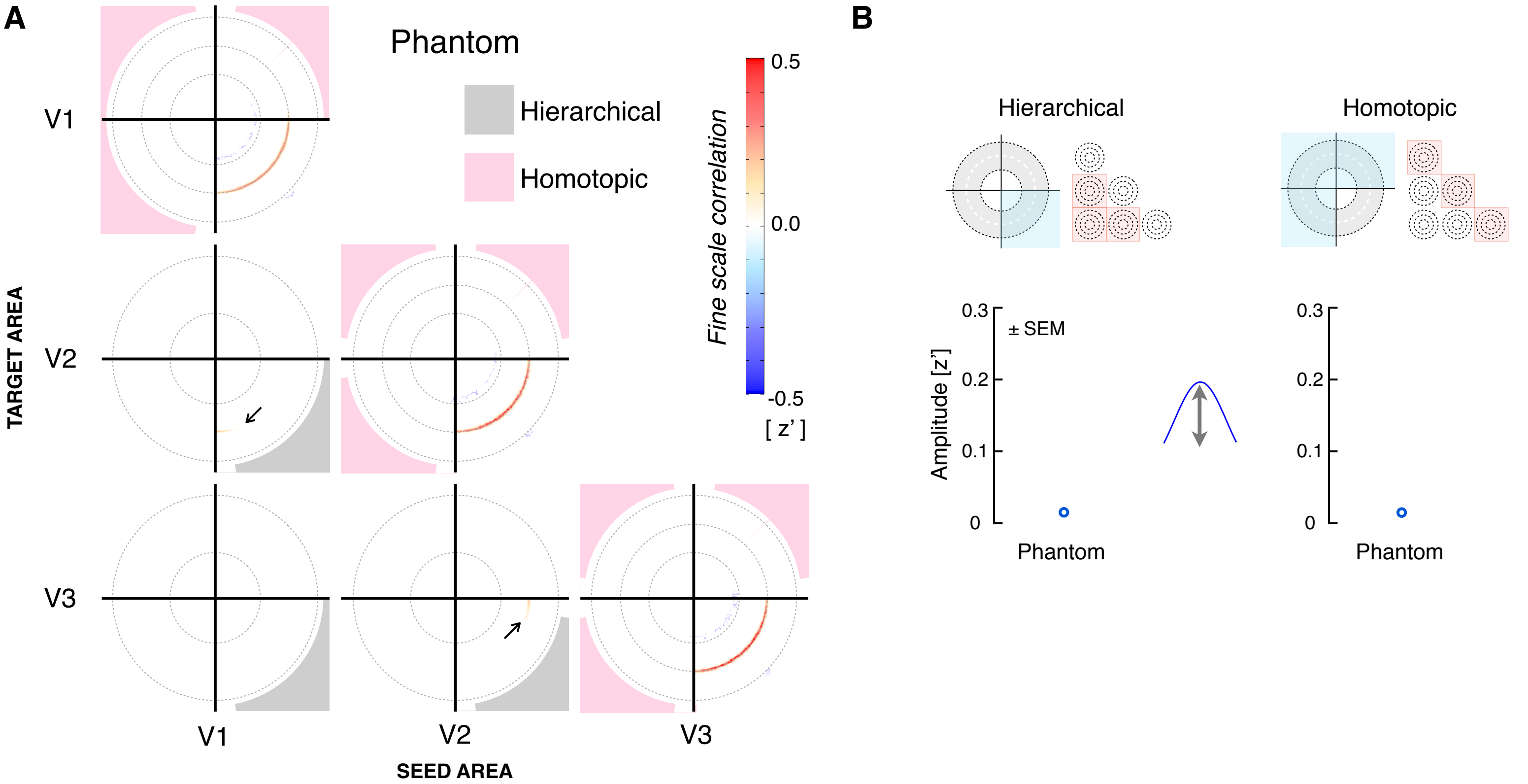

FIGURE S4 I Fine-scale radial symmetry data derived from phantom scans. (A) Phantom (n=8) radial symmetry plots between V1, V2, and V3. The raw time-series data from 8 random subjects were replaced with data from a water phantom scanned under otherwise identical conditions. Radial symmetry plots were then constructed as described in Figure 3. Local digital smoothness is visible in plots of a given visual area compared to self (V1-V1, V2-V2, V3-V3). The small arrows indicate an extension of local image smoothness to the "hierarchical" region of the plots for V1-V2 and V2-V3. The existence of this small correlation component motivated the exclusion of data from the borders of visual areas in the calculation of fine-scale correlation structure. **(B)** Fine-scale amplitude derived from phantom data. The central peak of the correlations for each of the quadrants was obtained and aggregated. We find that MR image correlation itself contributes no more than 0.02 to the Fisher z' values measured for the hierarchical, fine-scale correlation measure.
