# Supplementary material for: Hierarchical and homotopic correlations of spontaneous neural activity within the visual cortex of the sighted and blind
Source: Front Hum Neurosci. 2015 Feb 10;9:25. doi: 10.3389/fnhum.2015.00025 (PMC4322716; doi:10.3389/fnhum.2015.00025)
Supplement: Supplementary file 8 [file Image5.PDF]

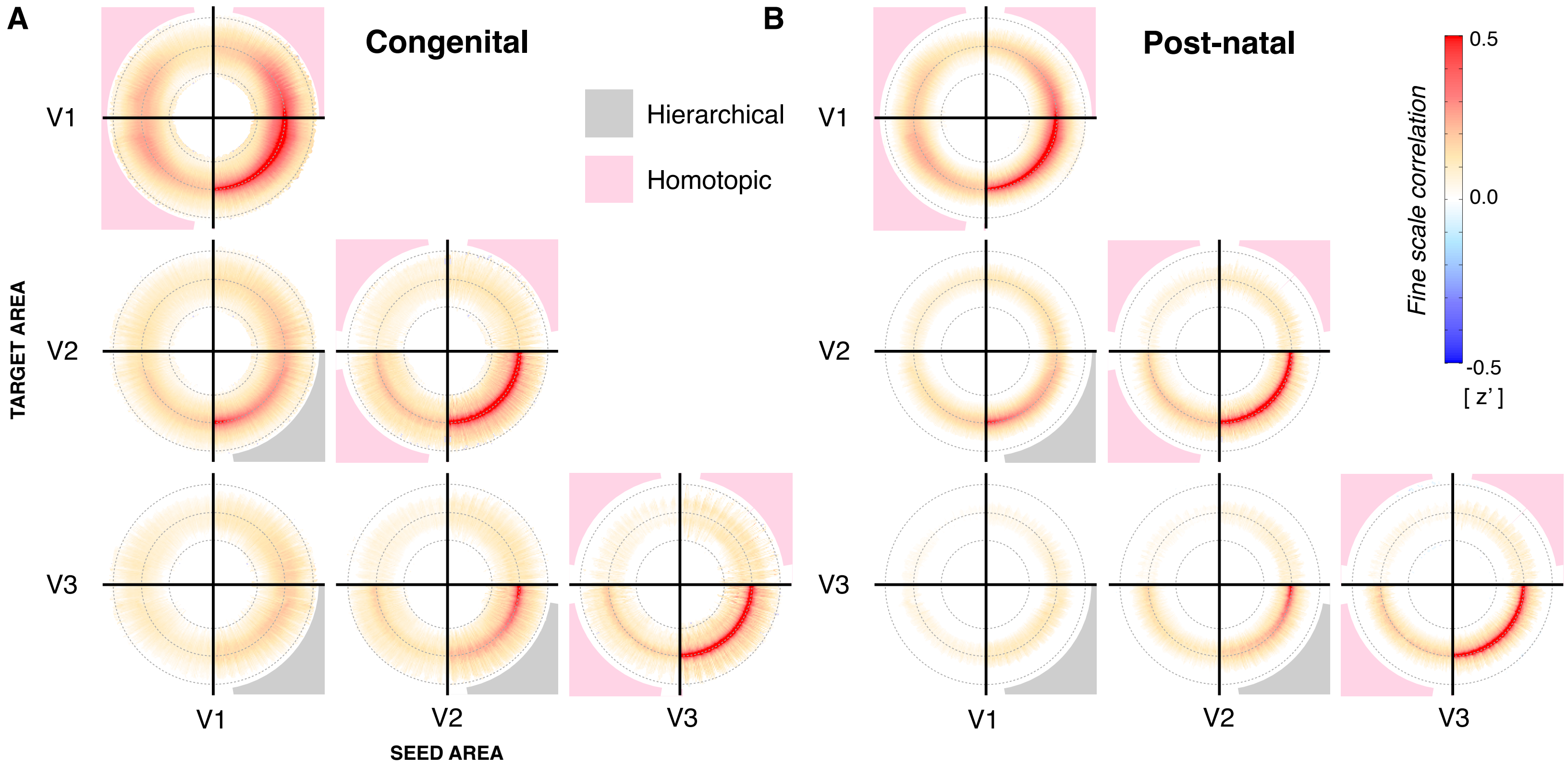

FIGURE S5 I Radial symmetry plots from the congenital and post-natal subgroups. (A) Average radial symmetry plots from congenitally blind subjects (n=14) between V1, V2, and V3. Similar to Fig. 3, the quadrants reflecting direct, hierarchical correlation are shaded in grey, and homotopic correlations shaded in pink. **(B)** Average radial symmetry plots from post-natal blind subjects (n=11).
