# Supplementary material for: Hierarchical and homotopic correlations of spontaneous neural activity within the visual cortex of the sighted and blind
Source: Front Hum Neurosci. 2015 Feb 10;9:25. doi: 10.3389/fnhum.2015.00025 (PMC4322716; doi:10.3389/fnhum.2015.00025)
Supplement: Supplementary file 1 [file Table1.PDF]

Table S1. Subject population demographics

ID	Sex / Age / Handedness			Age of onset	Light threshold (dB)	Cause
B05	F	23	R	0	-33.4	Congenital cataracts, detached retinas
B16	F	61	R	0	-16.1	Optic atrophy
B18	M	50	R	0	20.3	Congenital cataracts
B14	F	62	R	0	115.5	ROP
B24	M	62	L	0	122.8	Congenital cataracts; glaucoma (secondary)
B01*	F	33	L	0	LP	Congenital hypoplasia
B17	M	48	R	0	LP	ROP
B03	F	32	R	0	NLP	Retinopathy of prematurity (ROP)
B06	F	48	-	0	NLP	ROP; glaucoma; vitreous hemorrhage
B08	F	53	A	0	NLP	ROP
B10	M	58	R	0	NLP	ROP
B13	M	58	R	0	NLP	ROP
B21	M	69	R	0	NLP	RP; glaucoma (secondary); detached retinas
B25*	F	69	R	0	NLP	Congenital optic atrophy
B11	F	69	R	13	-32.7	Juvenile macular degeneration
B09	F	61	-	40	-14.6	RP (retinitis pigmentosa)
B15	M	40	R	36	-3.6	Glaucoma (primary); Cataracts (secondary)
B02	F	69	R	7	-2.9	Cone-rod dystrophy
B19	M	53	R	20	0.3	Congenital infection
B12	F	46	R	8	92.4	RP
B07	F	55	R	8	NLP	Uveitis
B23	M	48	R	12	NLP	Trauma
B22	F	61	R	14	NLP	Retinoblastoma; Left eye lost at age 3; right at 14
B04	M	59	R	28	NLP	Diabetic retinopathy
B20	F	63	R	54	NLP	Diabetic retinopathy; detached retina
S01	M	33	R			
S04	M	27	R			
S06	M	41	R			
S07	M	26	R			
S09	M	28	R			
S11	M	32	R			
S13	F	58	R			
S14	F	52	R			
S15	F	46	R			
S16	M	48	R			
S17	F	68	R			
S19	F	43	R			
S20	F	51	R			
S21	M	58	R			
S22	F	44	R			
S02	F	22	R			
S03	M	25	A			
S05	F	25	R			
S08	F	22	R			
S10	F	22	R			
S12	F	19	R			
S18	F	24	R			

Table S1 | Subject Population demographics. Twenty-five blind (B1–B25) and 22 sighted (S1– S22) individuals participated in the study. The blind population was heterogeneous with respect to age of onset and cause, although all had severe vision loss. R, Right-handed; L, left-handed; A, ambidextrous. Subjects with residual light perception (LP) were further tested for their minimum light sensitivity threshold, reported in dB (normal performance is < -35 db); NLP, no light perception; —, missing value. Excluded subjects due to poor model fit are denoted with (*). Subject labels are the same as that used in a prior report of these data (Butt et al., 2013).
