# Supplementary material for: Hierarchical and homotopic correlations of spontaneous neural activity within the visual cortex of the sighted and blind
Source: Front Hum Neurosci. 2015 Feb 10;9:25. doi: 10.3389/fnhum.2015.00025 (PMC4322716; doi:10.3389/fnhum.2015.00025)
Supplement: Supplementary file 2 [file Table2.PDF]

Table S2. Results Summary

	Regional [z']			Fine-scale [z']		[log (seed e° / target e°)]	
ID	Hierarchical	Homotopic	Background	Hierarchical Amplitude	Homotopic Amplitude	Hierarchical Sigma	Homotopic Sigma
B05	1.0577	0.5720	0.3340	0.3415	0.2051	0.6570	0.5989
B16	0.9409	0.7743	0.5578	0.2368	0.2400	0.7561	0.6847
B18	0.8822	0.7033	0.5581	0.2374	0.2141	0.5679	0.5697
B14	0.8161	0.4320	0.4446	0.1733	0.1240	0.6139	0.5077
B24	0.5937	0.7250	0.3854	0.2310	0.2283	0.3447	0.4672
B01	0.6560	0.6232	0.4605	-	-	-	-
B17	1.0095	0.4972	0.4885	0.2543	0.1627	0.4810	0.5193
B03	0.9031	0.4152	0.3657	0.2277	0.1331	0.3906	0.3731
B06	0.7615	0.7316	0.4680	0.2141	0.1867	0.5117	0.4749
B08	1.1471	0.9749	0.8188	0.4416	0.3907	0.9338	1.0722
B10	0.9794	0.6396	0.6176	0.2458	0.1833	0.5246	0.4793
B13	0.7711	0.8214	0.6011	0.2727	0.2877	0.7523	0.7514
B21	0.6827	0.3765	0.3340	0.1568	0.1080	0.5963	0.7723
B25	0.9337	0.4775	0.4627	0.2029	-	0.8265	-
B11	0.5826	0.5159	0.2536	0.1357	0.1279	0.4855	0.5870
B09	0.7512	0.7641	0.5133	0.2133	0.2181	0.5174	0.5456
B15	0.5915	0.7861	0.4567	0.1689	0.2229	0.4804	0.5524
B02	0.9820	0.7908	0.4183	0.4282	0.3270	0.3158	0.3100
B19	1.1093	0.7112	0.6271	0.2711	0.2096	0.4767	0.4899
B12	0.7886	0.2801	-0.0052	0.2179	0.1224	0.3805	0.3225
B07	0.9112	0.4172	0.3341	0.2818	0.1755	0.2455	0.1927
B23	0.4467	0.5265	0.2693	0.1408	0.1762	0.3506	0.3229
B22	0.5038	0.3709	0.2571	0.1110	0.0994	0.4582	0.3773
B04	0.4349	0.5662	0.1559	0.0884	0.1087	0.5110	0.7957
B20	0.8430	0.7335	0.4413	0.3159	0.2710	0.4010	0.4369
S01	0.7869	1.0298	0.5763	0.2615	0.3004	0.3706	0.4940
S04	0.7345	1.1362	0.5656	0.2428	0.2947	0.4276	0.5634
S06	0.6001	0.5388	0.2688	0.1285	0.1163	0.5738	0.8934
S07	0.6214	1.0935	0.3696	0.2504	0.3485	0.3550	0.4568
S09	1.2457	1.2509	0.9709	0.3428	0.3247	0.4227	0.4529
S11	0.3258	1.2537	0.2298	0.1340	0.3545	0.6154	0.7620
S13	0.5127	0.5236	0.2640	0.0672	0.0711	0.5709	0.5185
S14	1.2801	1.2282	1.1394	0.4613	0.4351	0.3746	0.3652
S15	0.8522	0.7824	0.5427	0.2718	0.2305	0.2565	0.3191
S16	0.8314	0.7191	0.4507	0.1931	0.1636	0.3539	0.4971
S17	0.7761	0.5967	0.4856	0.1520	0.1341	0.4452	0.4231
S19	0.3700	0.5610	0.1938	0.0519	0.0844	0.5342	0.7817
S20	0.2128	0.6397	0.0806	0.0676	0.1308	0.2028	0.7335
S21	0.5522	0.5840	0.3038	0.0908	0.0994	0.5217	0.4633
S22	0.6299	1.1196	0.5012	0.1712	0.3129	0.6079	0.7069
S02	0.5526	0.8229	0.3666	0.2396	0.2599	0.2367	0.3467
S03	0.6840	0.8864	0.5516	0.1383	0.1665	0.7183	0.6768
S05	0.5447	0.4847	0.3228	0.1258	0.0855	0.2776	0.5085
S08	0.5403	0.8463	0.3194	0.2573	0.2737	0.2157	0.3042
S10	0.7223	0.5771	0.4043	0.1748	0.1503	0.3717	0.3470
S12	1.7122	1.6170	1.3128	0.4243	0.3142	0.6535	0.6701
S18	0.6442	0.6720	0.4495	0.1779	0.2046	0.6953	0.6475

Table S2 | Regional and Fine-scale results summary. Regional results include direct hierarchical, homotopic, and background correlations. Fine-Scale results include individual subject amplitude and sigma fits for hierarchical and homotopic correlations. Excluded subjects due to poor model fit are denoted with (-).
